# The complete chloroplast genome of *Pleione hookeriana* (Orchidaceae) from Yunnan Province, China

**DOI:** 10.1080/23802359.2022.2040392

**Published:** 2022-02-17

**Authors:** Weiguo Chai, Xiang Chen, Huimin Li, Pengguo Xia

**Affiliations:** a Institute of Biotechnology, Hangzhou Academy of Agricultural Sciences, Hangzhou, China; b Key Laboratory of Plant Secondary Metabolism and Regulation of Zhejiang Province, College of Life Sciences and Medicine, Zhejiang Sci-Tech University, Hangzhou, China

**Keywords:** Chloroplast genome, endangered species, Orchidaceae, phylogenetic analyses, *Pleione hookeriana*

## Abstract

*Pleione hookeriana* (Lindl.) B. S. Williams is a species of Orchidaceae with high ornamental value. It is a protected plant in China. To document the genetic history of this rare species, the chloroplast genome sequence of *P. hookeriana* from the Yunnan Province, China, were analyzed. The complete chloroplast genome is 158,930 bp in length and contains a large single-copy (LSC) region of 86,880 bp, a small single-copy region of 18,664 bp (SSC), and a pair of inverted repeats (IR) of 26,693bp. There are 137 genes, including 89 protein-coding, 40 transfer RNA (tRNA) and 8 ribosomal RNA (rRNA) genes. The total GC content of the chloroplast genome sequence is 37.2%. The maximum-likelihood phylogenetic analysis indicated that *P. hookeriana* was sister to *P. chunii* (MK792342.1). The result may be because the species are roughly the same geographical location and advanced and developed from the same ancestor. This study provides important information for the identification and conservation of species, and genetic engineering of *P. hookeriana.*

*Pleione hookeriana* (Lindl.) B. S. Williams (*Pleione hookeriana* (Lindl.), Rollisson, William 1875) is an epiphytic herb that grows on tree trunks and moss-covered rocks or rock walls at the edge of shrubs. Distributed in the north of Guangdong, the west to the north of Guangxi, the southeast of Guizhou, the southeast of Yunnan and the south of Tibet between 1600 and 3100 meters above sea level. Due to the lack of extensive sampling and sufficient molecular evidence, the classification of *P.hookeriana* and *P. chunii* is still in a fuzzy state (Zhang et al. 2018). In recent years, the comparative analysis of the complete chloroplast genomes of different related species has provided promising methods for the study of phylogeny, population dynamics, and species evolution (Shaw et al. 2014; Jiang et al. 2019).

The research content and data reported in this paper are all conducted ethically and comply with relevant experimental and legislative guidelines. The collection of plant materials involved and the acquisition of data have been ethically approved by local agencies. Fresh leaves tissue of *P. hookeriana* were collected from Heqing County, Dali City, Yunnan Province (26°28'35″N, 100°25'32"E). The voucher specimen was preserved at HAAS (Hangzhou Academy of Agricultural Sciences, http://www.hznky.com) in charge of Weiguo Chai (Voucher number: Weiguo Chai et al 2103005 and Kuni@21cn.com).

Total genomic DNA was extracted using a modified CTAB method. The DNA was deposited at the Key Laboratory of Plant Secondary Metabolism and Regulation in Zhejiang Province, Zhejiang Sci-Tech University (http://sky.zstu.edu.cn) under the voucher number ZSTUX0201 (collected by *Pengguo Xia* and xpg_xpg@zstu.edu.cn). The sequencing library was constructed by the Illumina Hiseq^TM^ platform with an insert size of about 400 bp. In total, Raw PE reads were generated and approximately 2.508 Gb of clean PE reads were obtained after removing adapter and low-quality bases. NOVOPlasty v2.7.2 (Dierckxsens et al. 2017) was used to assemble the complete chloroplast genome of *P. hookeriana* with default settings. The sequence was annotated using geneious Prime. The complete chloroplast genome of *P. hookeriana* was obtained and submitted to GenBank (Accession number MZ958823). The complete chloroplast genome sequence of *P. hookeriana* is 158,930 bp in length and contains a LSC of 86,880 bp, a SSC of 18,664 bp and a IRs of 26,693 bp. There are 137 genes, including 89 protein-coding, 40 transfer RNA (tRNA) and 8 ribosomal RNA (rRNA) genes. The total GC content of the chloroplast genome sequence is 37.2%. Compared with the structures of *P. pleionoides*, *P. forrestii*, *P. chunii*, *P. maculata* and *P. formosana*, *P. hookeriana* has two more tRNAs. The GC content of genus *Pleione* are 37.3% except *P. maculata,* which is 37.2% (Jiang et al 2019; Chen et al 2019; Wu, Shen, et al 2019; Wu, Wu, et al 2019; He et al 2021).

The phylogenetic analysis of the *P. hookeriana* complete chloroplast genome and 21 related species was constructed using the best-selected TVM + F+R4 model and 1000 bootstrap replicates with IQTREE V1.6.7 (Nguyen et al.^2015^). The maximum-likelihood phylogenetic analysis indicated that *P. hookeriana* was sister to *P. chunii* (MK792342.1) (Figure 1). *P. chunii* is regarded by some authorities as a variant of *P. hookeriana*. In fact, they are all epiphytic plants with very similar lip shapes and flower colors. However, *P. chunii* has obvious pseudobulbs, larger flowers, and callus on the lips (). The maximum-likelihood phylogenetic analysis indicated that *P. chunii* was sister to *P. bulbocodioides* and *P. formosana* (Wu SS et al. 2019). A similar situation appeared in our evolutionary analysis, from which we can conclude that *P. hookeriana* was sister to *P. chunii* (MK792342.1).

**Figure 1.**
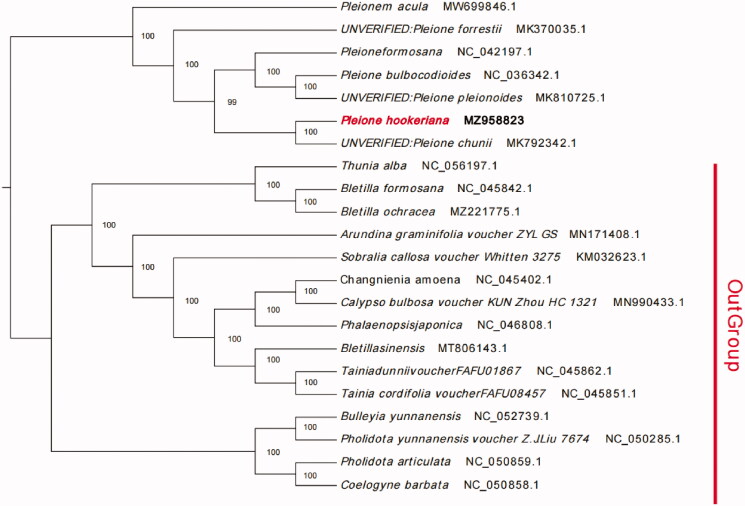
Maximum likelihood (ML) tree of *P. hookeriana* and its related relatives, including 6 Pleione species based on the complete chloroplast genome sequences.

## Data Availability

The data that support the findings of this study are openly available in NCBI (https://www.ncbi.nlm.nih.gov) GenBank with the accession number (MZ958823). The associated BioProject, SRA and BioSample numbers are PRJNA755890, SRR15524497, and SAMN20856062 respectively.
